# *Dominant-negative NARS1 R534∗* mutation causes wild-type subunit poisoning and heterodimer predominance in cells

**DOI:** 10.1016/j.jbc.2025.110690

**Published:** 2025-09-04

**Authors:** Ingrid Vallee, Ryan Shapiro, Leo Qi, Marisa I. Mendes, Desiree E.C. Smith, Qian Zhang, Taisuke Kanaji, Bernhard Kuhle, Xiang-Lei Yang

**Affiliations:** 1 Department of Integrative Structural and Computational Biology, The Scripps Research Institute, La Jolla, California, USA; 2 Department Laboratory Medicine, Laboratory Genetic Metabolic Diseases, Amsterdam UMC, University of Amsterdam, Amsterdam, The Netherlands; 3 Amsterdam Gastroenterology Endocrinology Metabolism, Amsterdam UMC, University of Amsterdam, Amsterdam, The Netherlands; 4 Institute of Systems and Physical Biology, Shenzhen Bay Laboratory, Shenzhen, China; 5 Department of Cellular Biochemistry, University Medical Center Göttingen, Göttingen, Germany; 6 Research Group Structure and Function of Molecular Machines, Max Planck Institute for Multidisciplinary Sciences, Göttingen, Germany

**Keywords:** aminoacyl-tRNA synthetase, NARS1, AsnRS, asparaginyl-tRNA synthetase, enzyme, heterodimer, mutation

## Abstract

Aminoacyl-tRNA synthetases (aaRSs) catalyze the aminoacylation of tRNA with their cognate amino acids, an essential step in protein biosynthesis. While biallelic mutations in aaRSs often result in severe multi-organ dysfunction accompanied by developmental delays, monoallelic mutations typically cause milder, tissue-specific symptoms. However, a *de novo* monoallelic nonsense mutation (R534∗) in the asparaginyl-tRNA synthetase (AsnRS)—resulting in a premature stop codon and 15-residue C-terminal truncation—has been identified in multiple families and is associated with severe neurodevelopmental symptoms. Here, we find that patient-derived lymphoblasts express similar amounts of wild-type (WT) and mutant (R534∗) AsnRS and exhibit a severe proliferation defect. Like most aaRS family members, AsnRS functions as a homodimer. Structural analysis indicates that the region deleted in AsnRS^R534∗^ (R534-P548) contributes to dimerization, tRNA binding, and stabilization of the catalytic site architecture. Indeed, AsnRS^R534∗^ forms a weaker homodimer than AsnRS^WT^, displays impaired tRNA binding, along with a severe loss of enzymatic activity. Nevertheless, when exogenously expressed in human cells, AsnRS^R534∗^ shows a much stronger tendency than AsnRS^WT^ to dimerize with the endogenous WT enzyme, driving R534∗/WT heterodimer predominance in the cell. Notably, the heterodimer is severely defective in enzymatic function, comparable to the AsnRS^R534∗^ homodimer, indicating that AsnRS^R534∗^ exerts a dominant-negative loss-of-function effect on the WT subunit through heterodimerization. These findings provide a mechanistic explanation for how a monoallelic AsnRS mutation can lead to profound cellular dysfunction and contribute to severe neurodevelopmental disease, offering new insights into aaRS-associated pathologies and potential therapeutic strategies.

Aminoacyl-tRNA synthetases (aaRSs) are a family of ancient and highly conserved enzymes that play a central role in protein biosynthesis by catalyzing the aminoacylation of tRNA for subsequent use by the ribosome (1). Mutations in aaRSs cause a wide range of diseases (2). Biallelic recessive mutations typically impair enzyme activity, resulting in a loss of function and leading to severe, early-onset multi-organ disorders (2). By contrast, monoallelic mutations are often associated with late-onset diseases of milder severity and tissue specificity, such as the peripheral neuropathy Charcot-Marie-Tooth disease (3). Monoallelic mutations do not always impair enzyme activity (4, 5, 6, 7), and when they do, the loss of function in the mutant allele may not manifest in the presence of a wild-type (WT) allele in patient cells (8).

Recent reports have linked both mono- and bi-allelic mutations in the human asparaginyl-tRNA synthetase (AsnRS or *NARS1*) to impaired enzymatic function and severe diseases (9, 10, 11). Among them, the mono-allelic nonsense mutation R534∗ and bi-allelic missense mutation R545C are the most recurrent mutations arising in multiple independent families. The R534∗ nonsense mutation results in a premature stop codon in the last exon of the *NARS1* gene and a 15-residue truncation at the C-terminus (ΔR534-P548) of the AsnRS protein. The patients exhibit a spectrum of neurodevelopmental abnormalities, including developmental delay, microcephaly, intellectual disability, ataxia, and seizures, as well as peripheral neuropathy, with onset of disease at birth for the mono-allelic R534∗ mutation and in childhood for the bi-allelic R545C mutation. *AsnRS activity is more severely reduced in patient cells with the mono-allelic R534*∗ mutation than in those with the bi-allelic R545C mutation (10). Notably, the R534∗ mutation is the first reported aaRS monoallelic mutation causing a severe loss of function in patient cells.

The aaRS-catalyzed aminoacylation reaction is carried out in two steps. In the first step, aaRSs bind to an amino acid and adenosine 5′-triphosphate (ATP) to catalyze the formation of an enzyme-bound aminoacyl-adenylate. The reaction is driven by the energy released from ATP hydrolysis, which generates pyrophosphate (PPi). In the second step, the activated amino acid reacts with tRNA to form aminoacyl-tRNA. Most members of the aaRS family, especially class II tRNA synthetases, including AsnRS, function as homodimers. Dimerization *via* the catalytic domain stabilizes the structure and shapes the active site architecture to support efficient binding of ATP, amino acid, and tRNA (3). Many dimeric tRNA synthetases exhibit half-site reactivity and allosteric inter-subunit communication, where substrate binding in one subunit induces conformational changes in the other to enhance the fidelity and efficiency of tRNA charging (12, 13, 14, 15, 16, 17, 18).

The severe loss of AsnRS function observed in *patient cells carrying the mono-allelic R534*∗ mutation suggests that this mutation may exert a dominant-negative effect on the WT enzyme. To exhibit strong dominant-negative interference in a dimeric enzyme, the following conditions must be met: (1) The mutant must be stably expressed to exert its effect, (2) the mutant subunit should preferentially form heterodimers with the WT enzyme rather than assembling into mutant homodimers or allowing WT homodimers to predominate, and (3) the activity of the heterodimer must be severely reduced compared to the WT homodimer.

The three-dimensional structure of human AsnRS (19, 20) closely resembles that of class IIb AspRS (21) and LysRS-II (22), with an idiosyncratic N-terminal appended domain, followed by the evolutionarily conserved anticodon binding domain and the C-terminal catalytic domain (Fig. 1*A*). The N-terminal domain, UNE-N, is eukaryotes specific and does not exist in prokaryotic AsnRS (23). The UNE-N domain may help to anchor the synthetase onto the tRNA (24) but may also have non-enzymatic functions (19). *Both the R534∗* and R545C mutations are located in the C-terminal catalytic domain of AsnRS, which contains the active site formed by a six-stranded antiparallel β-sheet and three highly conserved motifs essential for aminoacylation (25) (Fig. 1*A*).

**Figure 1:**
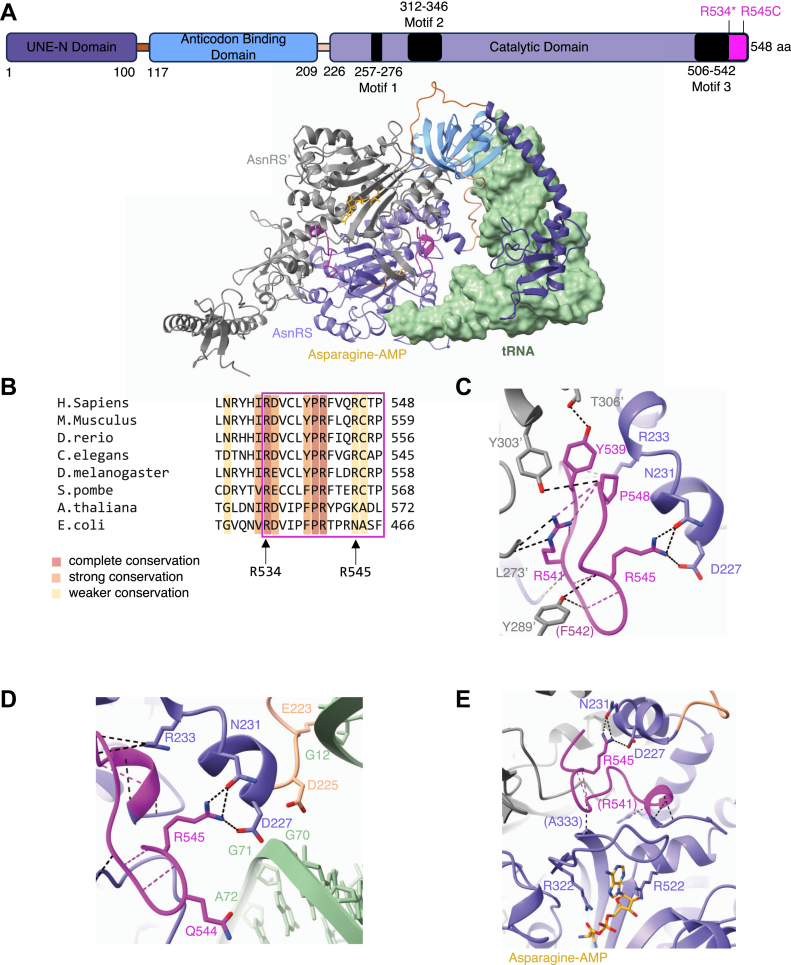
**Structural analysis suggests R534∗ may impact dimerization, tRNA binding, and catalytic activity.***A*, domain structure of *Homo sapiens* AsnRS and the AlphaFold3 model of AsnRS in complex with tRNA^Asn^-GTT. The tRNA^Asn^ (*green*) is predicted to bind one subunit of the AsnRS homodimer *via* the anticodon binding domain (*light blue*), the catalytic domain (*purple*) and the UNE-N domain (*dark purple*). The R534-P548 deletion (*pink*) in R534∗ is located at the C-terminus of the enzyme. The second AsnRS subunit (AsnRS’) is represented in *gray*. *B*, sequence alignment of the AsnRS C-terminus across the domains of life. R534 is conserved throughout evolution, R545 is conserved in most eukaryotes. *C*, the R534-P548 region is involved in dimerization. Residues Y539, R541, F542, and R545 stabilize the dimer interface between the two subunits by H-bonds. R545 forms stabilizing H-bonds within the same subunit. *D*, R545 stabilizes the loop adjacent to the tRNA acceptor stem (G70) and D loop (G12). *E*, residues in the R534-P548 loop stabilize the active site *via* interactions affecting key residues involved in substrate recognition and catalysis (*e.g.*, R322 and R522 in motifs 2 and 3, respectively). Residues in parenthesis form interactions *via* their backbone atoms.

In this study, we investigated the functional impact of the R534∗ and R545C mutations on AsnRS activity. Moreover, we *examined the effect of R534∗* on the dimerization behavior of AsnRS, both in homodimers and in heterodimers with the WT enzyme, both *in vitro* and in mammalian cells. Our findings reveal a differential impact of the mutation on homodimer and heterodimer stability, *with R534∗/*WT heterodimers predominating in cells, as well as a dominant-negative effect in which the mutant subunit compromises the function of the WT subunit. These results support a toxic mechanism for the R534∗ mutation, highlighting its pathogenic role.

## Results

### Structure analysis suggests R534∗ may impact dimerization and catalytic activity

To predict the impact of the R534∗ mutation and resulting ΔR534-P548 deletion on enzymatic activity, dimerization, and tRNA binding, we analyzed the crystal structures of WT AsnRS (19, 20) and generated a model of AsnRS in complex with tRNA^Asn^-GTT using AlphaFold 3 (Fig. 1*A*). According to these structures, R534-P548 forms a short α-helix followed by a hairpin loop at the C-terminus of the protein (Fig. 1*A*). Most residues within the last 15 amino acids are evolutionarily well-conserved (Fig. 1*B*). R534 is strictly conserved and R545 is conserved in most eukaryotes. As revealed by the crystal structure (PDB 8H53) (20), multiple residues within the C-terminal stretch participate in dimerization as they form hydrogen bonds with residues of the other subunit (Fig. 1*C*). Namely, the side chains of Y539 and R541, as well as the backbone of F542 and P548 from subunit one engage in H-bonding interactions with residues T306′, Y303′, L273′ and Y289′ from subunit 2 (gray). Moreover, the R534-P548 region is located near the acceptor stem of tRNA^Asn^ and may contribute indirectly to tRNA binding by stabilizing adjacent residues (Fig. 1*D*). Finally, residues R534-P548 are positioned near the active site where they form hydrogen bonds with residues from motifs two and 3. Consequently, the R534∗ mutation could alter the active site architecture, affecting key residues involved in substrate recognition and catalysis - such as the “arginine tweezer” R322 and R522 (26) located in motifs two and 3, respectively (Fig. 1*E*). Interestingly, the R545 side chain points away from the dimer interface (Fig. 1*C*) and the active site (Fig. 1*E*) to interact with residues D227 and N231 within the same subunit (purple) near the N-terminal end of the catalytic domain. Disruption of these interactions by the R545C mutation would thus likely result in local destabilization (Fig. 1*E*). Taken together, this model highlights the critical location of the C-terminal region and suggests that the R534∗ mutation could influence dimerization and enzymatic activity, while the single point mutation R545C should have a milder impact.

### The R534∗ mutant shows dominant toxicity in cells

**Figure 2:**
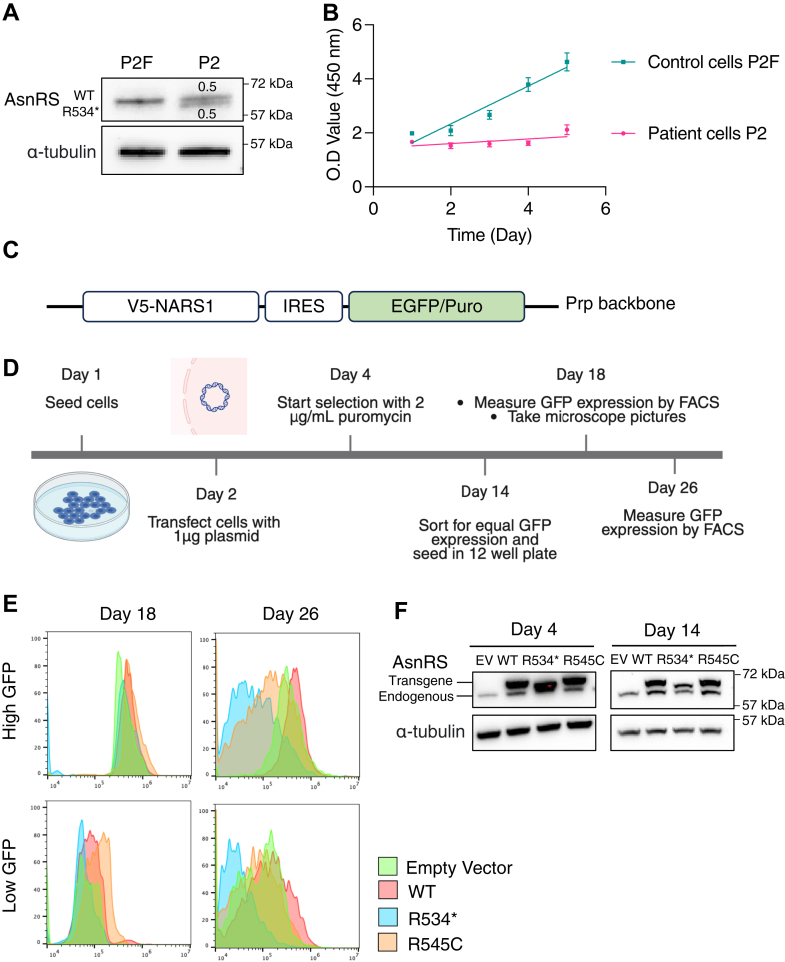
**The R534∗ mutant shows dominant toxicity in cells.***A*, Western blot analysis of AsnRS expression in lymphoblasts obtained from patient P2 and the patient’s healthy father (P2F) with Western blot quantification of AsnRS signal normalized over alpha-tubulin signal. N = 1. *B*, CCK8 cell viability assay quantifying the number of live cells over the course of 5 days in culture. N = 4 technical replicates. *C*, plasmid construct to achieve proportional expression of V5-AsnRS and GFP. *D*, timeline of cell transfection and selection workflow. *E*, FACS analysis of GFP signal to indicate expression of AsnRS variants after transient transfections in HEK293T cells. Measurements at day 18 and day 26 after transfection (day 3 and 11 post FACS), respectively. N = 1. *F*, Western blot analysis of AsnRS expression in HEK293T cell on Day 4 and Day 14 after puromycin selection following transient transfections. N = 3 biological replicates.

Patient P2 is heterozygous for the truncated R534∗ variant. According to a previous analysis, patient P2 lymphoblasts contain overall *NARS1* mRNA levels similar to those in cells from a healthy parent (P2F) (10). However, this analysis did not distinguish the expression of the WT *versus* the R534∗ allele. To confirm equal expression at the protein level, we visualized the AsnRS protein by Western blot. The patient cells display two bands of similar intensity, accounting for the full length and Δ534 to 548 AsnRS (Fig. 2*A*).

We noted an extremely slow growth of the P2 patient cells in culture compared to the parental P2F cells (Fig. 2*B*). To confirm the impact of the R534∗ mutation on cell proliferation, we overexpressed *NARS1* variants in HEK293T cells. After puromycin selection, we used FACS to sort cells based on GFP signal, which indicates the level of AsnRS overexpression (Fig. 2, *C* and *D*). High AsnRS^WT^- and AsnRS^R545C^-expressing cells grew at a similar speed to each other but slower compared to the empty vector control (Fig. S1*A*). Interestingly, the morphology of the high AsnRS^WT^-expressing cells was also different from the empty vector control cells (Fig. S1*B*), suggesting that overexpression of WT AsnRS itself has an impact, as previously seen in zebrafish (10). By far the strongest growth defect was observed for high AsnRS^R534∗^-expressing cells, which grew slower compared to high AsnRS^WT^- and AsnRS^R545C^-expressing cells. After 4 and 12 days in culture post-FACS sorting (Days 18 and 26, respectively), cells were re-analyzed by FACS. GFP expression significantly reduced in AsnRS^R534∗^- *versus* AsnRS^WT^-expressing cells for both high- and low-GFP groups, indicating that cells with lower levels of AsnRS^R534∗^ expression had a selective advantage over those with higher expression levels (Fig. 2*E*). Similarly, a different batch of transfected cells under puromycin selection showed a time-dependent decrease in expression only for the AsnRS^R534∗^ mutant, whereas the expression of AsnRS^WT^ and AsnRS^R545C^ remained stable (Fig. 2*F*). Therefore, we confirmed that the R534∗ mutant exhibits dominant toxicity, impairing cell proliferation in mammalian cells.

### R534∗ and R545C mutants are stable *in vitro* but have differential functional impact

**Figure 3:**
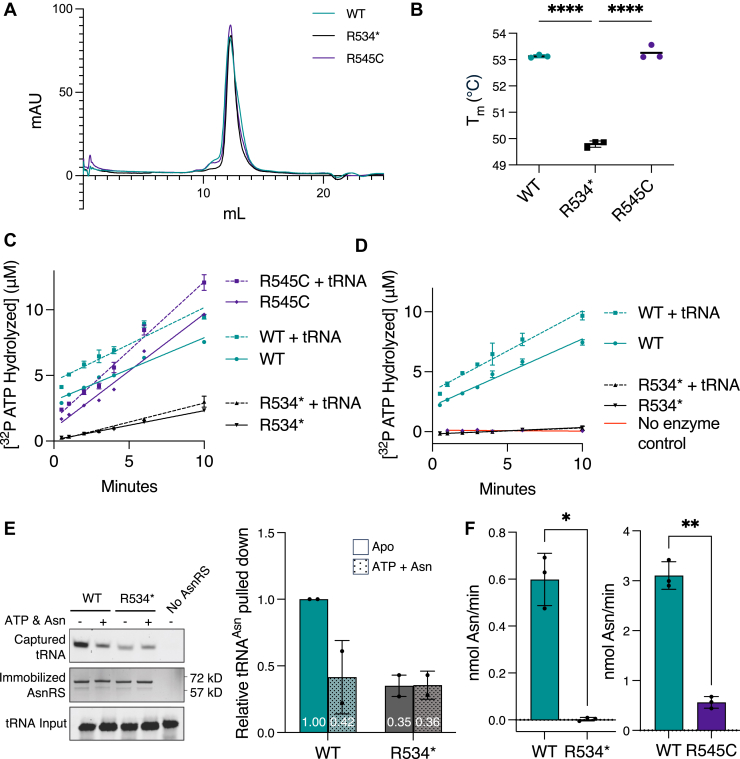
***In vitro* characterization of WT, R534∗, and R545C AsnRS**. *A*, gel filtration profiles of recombinant R534∗, R545C and WT AsnRS. *B*, melting temperatures (Tm) for WT, R534∗, and R545C AsnRS as determined by thermal shift assays. N = 3 biological replicates. Statistical significance was calculated by One-way ANOVA with Tukey correction (∗∗∗∗ *p*-value<0.0001). *C* and *D*, ATP hydrolysis activity of recombinant WT and R534∗ AsnRS (*C*: 10 μM AsnRS without hydroxylamine treatment; *D*: 8 μM AsnRS with hydroxylamine treatment) with and without *in vitro* transcribed tRNA^asn^ GTT-2 (at equal concentrations to the enzyme). N = 3 technical replicates. *E*, Ni-NTA pull-down assay to assess the binding of *in vitro* transcribed tRNA^asn^ GTT-2 by WT and R534∗ AsnRS in the presense or absence of Asn and ATP. N = 2 biological replicates. Representive images and quantification analysis are shown. Each data point represents mean ± standard deviation. *F*, aminoacylation activity of 1.5 μM WT or R534∗ AsnRS on yeast total tRNA after 10 min incubation time (*left*) and of 375 nM WT or R545C AsnRS after 3 min incubation time (*right*). Statistical significance was determined by unpaired *t* test with Welch’s correction (∗*p*-value = 0.0113, ∗∗*p*-value = 0.0012). N = 3 technical replicates.

The similar levels of WT and R534∗ AsnRS in cells from patient P2 suggests that the mutant protein is stable (Fig. 2*A*). For further *in vitro* analysis, we purified recombinant WT, R534∗, and R545C AsnRS. All three variants appear to form homodimers when analyzed by size exclusion chromatography (Fig. 3*A*). The *K*_d_ of dimerization was estimated using mass photometry (Fig. S2). The *K*_d_ of AsnRS^WT^ and AsnRS^R545C^ were similar (*K*_d_^WT^ = 3.0 ± 1.2 nM, *K*_d_^R545C^ = 5.2 ± 1.0 nM), confirming that the R545C mutation has little impact on dimerization. As expected from the structural analysis, the *K*_d_ of the AsnRS^R534∗^ mutant was significantly increased (*K*_d_^R534∗^ = 44.5 ± 10.2 nM; ∼15-fold), confirming that the deletion destabilizes homodimer formation. Moreover, the thermal stability of the R534∗ mutant (Tm=49.9 °C), as measured by thermal shift assay, was reduced compared to WT (Tm=53.1 °C) and R545C (Tm=53.3 °C) (Fig. 3*B*), correlating with the stability of the homodimers.

Next, we investigated the impact of the mutations on AsnRS enzymatic activity. We measured the ATP hydrolysis activity, which monitors the first step of the aminoacylation reaction, either in the absence or presence of an *in vitro* transcribed tRNA^asn^-GTT substrate (Fig. 3*C* and Fig. S3*A*). The WT enzyme showed the highest initial activity, indicating the amount of available active sites at 31%, represented by the Y-intercept of the simple linear regression fitted from the data points. The amount of available active sites was further increased to 47% upon the addition of tRNA. However, the addition of tRNA did not have a significant impact on the rate of ATP hydrolysis at steady state, represented by the slope of the simple linear regression, indicating a lack of impact of tRNA on turnover for the WT enzyme, as expected for some class II aaRS (1). By contrast, virtually no active sites were measured for the R534∗ mutant either with or without tRNA, indicating a severe loss of catalytic function for the R534∗ mutant. The R545C mutant showed a lower initial activity but a higher turnover rate than the WT, and both activities (initial and steady state) were modestly enhanced upon tRNA addition, suggesting that R545C has a much milder impact on the active site than the R534∗ mutation.

Because we observed varying degrees of low steady-state ATP hydrolysis activity (as indicated by the slope in Fig. 3*C*) across different batches of the R534∗ mutant, we speculated that the purified recombinant enzymes may contain differing amounts of bound Asn-AMP, the aminoacylation intermediate, which could contribute to the observed activity. To test this, we treated both WT and R534∗ proteins with hydroxylamine during purification to remove the adenylate (27). Hydroxylamine treatment completely abolished the activity of R534∗ (Fig. 3*D* and Fig. S3*B*), supporting the idea that the observed low steady-state ATP hydrolysis activity of R534∗ is attributable to pre-bound Asn -AMP and that the R534∗ mutant has some residual activity when expressed in *Escherichia coli* cells.

To further investigate the loss of function caused by the R534∗ mutation, we studied tRNA binding using *in vitro* transcribed tRNA^asn^-GTT and hydroxylamine-treated AsnRS proteins. Compared to the WT protein, the R534∗ mutant exhibited reduced tRNA binding (Fig. 3*E*). Interestingly, the addition of ATP and asparagine (Asn) led to decreased tRNA binding by the WT protein, presumably due to the release of aminoacylated tRNA. In contrast, no such decrease was observed for the R534∗ mutant, consistent with its impaired catalytic activity. Indeed, the R534∗ mutant displayed a severe loss of tRNA aminoacylation activity, whereas the R545C mutant retained partial activity relative to WT (Fig. 3*F*).

### R534∗ exhibits a dominant negative effect through heterodimerization with WT AsnRS

**Figure 4:**
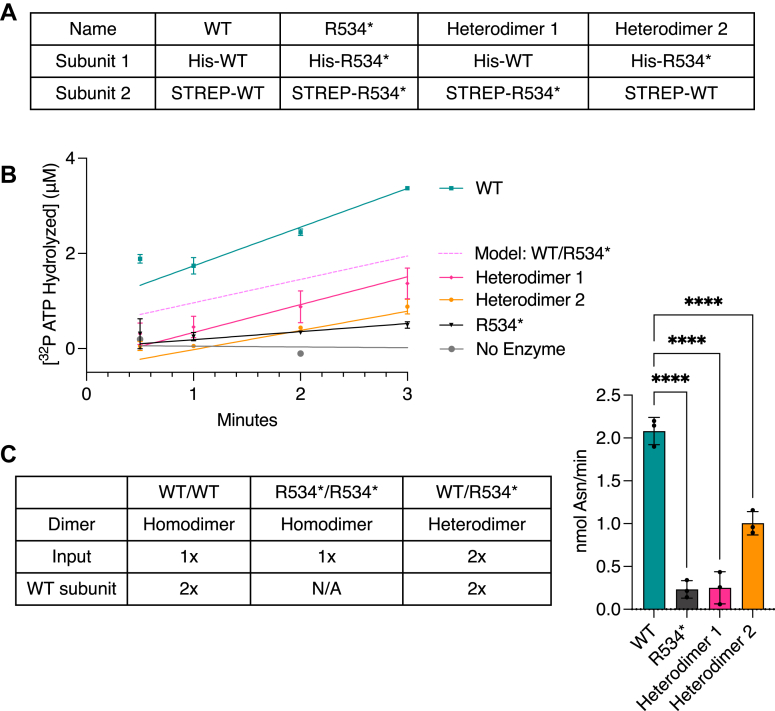
**The R534∗ mutant inhibits the WT subunit by allosteric regulation**. *A*, summary of purified recombinant constructs of WT and R534∗ homodimers and WT/R534∗ heterodimers. *B*, ATP hydrolysis activity of WT, R534∗ homodimers and WT/R534∗ heterodimers at 7 μM. The model activity of WT/R534∗ was calculated (WT + R534∗)/2. *C*, aminoacylation activity of 375 nM WT and R534∗ homodimers and 750 nM WT/R534∗ heterodimers after 3 min of reaction on yeast total tRNA. Results were compared using a One-way ANOVA statistical test (∗∗∗∗*p*-value<0.0001). N = 3 technical replicates.

We have shown that the R534∗ mutant weakens homodimer formation and causes a severe loss of function (Fig. 3, *D*–*F*). The central question remains for heterodimer formation and activity. Does the deletion of 15 amino acids also weaken the interaction between the mutant and a WT subunit? Does the loss of function in R534∗ poison the WT subunit *via* heterodimerization? In an attempt to recapitulate the effect of heterodimerization, we mixed separately purified WT and R534∗ enzymes and measured the activity. The activity of the mixture was equal to the average of the activities of WT and R534∗ measured separately (Fig. S3*C*), suggesting a lack of subunit exchange between the homodimers. Therefore, we established a protocol to co-express the WT and the R534∗ mutant with different affinity tags to allow isolation of heterodimers His-WT/STREP-R534∗ (1) and His-R534∗/STREP-WT (2, 28). To ensure that the tags do not differentially affect AsnRS activity, we purified His-WT and STREP-WT homodimers and confirmed their equal tRNA aminoacylation activity (Fig. S3*D*). As controls for the heterodimers, we also purified the His-WT/STREP-WT and His-R534∗/STREP-R534∗ homodimers (Fig. 4*A*).

The initial ATP hydrolysis activities of the heterodimers (both Heterodimer one and Heterodimer 2) were virtually identical to that of the R534∗ homodimers, suggesting a strong dominant negative effect of R534∗ on the WT subunit’s active site (Fig. 4*B*). Interestingly, the recombinant heterodimers exhibited increased steady-state activity compared to the R534∗ homodimer, suggesting either residual activity from the WT subunit or potentially elevated levels of pre-bound Asn-AMP, as these proteins were not subjected to hydroxylamine treatment (Fig. 4*B*). Next, we measured the tRNA aminoacylation activity in the heterodimers. *To directly assess the dominant negative effect of R534*∗ on the WT subunit, we doubled the heterodimer concentration relative to WT homodimers, ensuring the same amount of WT subunits was used in the assay. Notably, both heterodimer variants exhibited severely reduced activity, ranging from 10% to 50% of the WT activity, even at double concentrations (Fig. 4*C*). (Again, because these proteins were not subjected to hydroxylamine treatment, the variability in heterodimer activity may reflect differing amounts of pre-bound Asn-AMP.) In summary, our results demonstrate *in vitro* that the R534∗ mutant exhibits a dominant negative loss-of-function effect on the WT subunit through heterodimerization.

### The R534∗/WT heterodimer is more stable than WT and R534∗ homodimers in the cell

**Figure 5:**
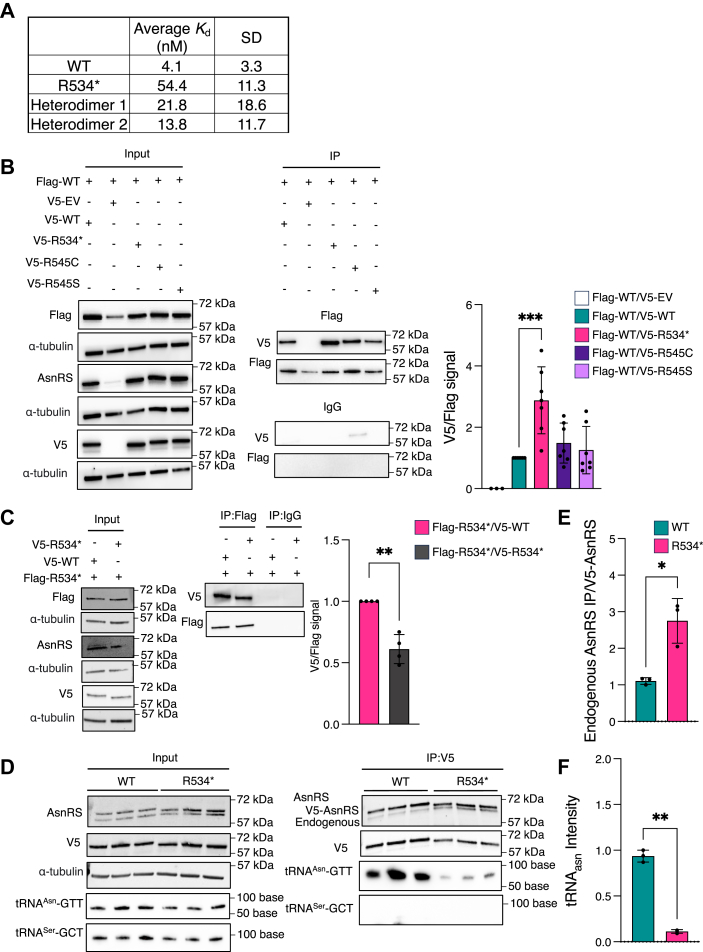
**The R534∗/WT heterodimer is the predominant form of AsnRS in the cell.***A*, *K*_d_ of dimerization for AsnRS WT and R534∗ homodimers and WT/R534∗ heterodimers measured by mass photometry. N = 2 biological replicates. *B*, immunoprecipitation of Flag-AsnRS in HEK293T cells transiently co-expressing Flag-AsnRS WT with different V5-AsnRS variants (EV (empty Vector), WT, R534∗, R545C, R545S). N = 7 biological replicates. One-Way ANOVA statistical test (∗∗∗*p*-value = 0.0002). *C*, immunoprecipitation of Flag-AsnRS in HEK293T cells transiently co-expressing Flag-AsnRS R534∗ with different V5-AsnRS variants (WT or R534∗). N = 4 biological replicates. Unpaired *t* test with Welch’s correction (∗∗*p*-value = 0.0071). *D*, co-immunoprecipitation of V5-AsnRS in HEK293T cells overexpressing R534∗ or WT at concentrations comparable to endogenous AsnRS. *E*, Quantification of the amount of the endogenous AsnRS pulled down by R534∗ or WT V5-AsnRS. Unpaired *t* test with Welch’s correction (∗*p*-value = 0.0404). N = 3 technical replicates. *F*, quantification of the amount of endogenous tRNA^asn^ GTT-2 bound to R534∗ or WT V5-AsnRS. Unpaired *t* test with Welch’s correction (∗∗*p*-value = 0.0010). N = 3 technical replicates.

To understand how R534∗ affects heterodimer formation, we next determined the *K*_d_ of dimerization for the purified heterodimers. Interestingly, the *K*_d_ for both heterodimers was in-between those of the WT and R534∗ homodimers but closer to that of the WT (Fig. 5*A*). (The WT and R534∗ homodimers exhibit *K*_*d*_s similar to those measured for the His-tagged homodimers.) Although this result suggests a stronger capacity of the R534∗ mutant to form R534∗/WT heterodimers than R534∗/R534∗homodimers, the WT/WT homodimer would still be the predominant form of dimers if R534∗ and WT AsnRS are expressed equally, which would not explain the strong dominant negative effect we see at the activity level.

Therefore, we turned to a cell-based approach to study heterodimerization. Flag-tagged WT and V5-tagged WT and mutant AsnRS constructs were transiently overexpressed in HEK293T cells followed by co-immunoprecipitation to measure the levels of interaction between WT and mutant variants (Fig. 5*B*). Remarkably, a significantly larger amount of V5-R534∗ than V5-WT was pulled down with Flag-WT AsnRS with anti-Flag antibodies, indicating a more stable WT/R534∗ heterodimer interaction than WT/WT homodimer interaction. By contrast, the amount of V5-R545C pulled down by Flag-WT AsnRS was similar to that of V5-WT (Fig. 5*B*), confirming that the R545C mutation does not affect dimerization, either as purified homodimer or as a WT/R545C heterodimer in cells (Fig. 5*B*). Because the R545C mutant consistently showed binding to the IgG control, we tested an alternative substitution, R545S, which did not bind to the IgG control and did not affect dimerization either (Fig. 5*B*), further supporting that R545 is not involved in dimerization. Moreover, we transiently expressed Flag-tagged R534∗ together with V5-tagged WT or R534∗ variants (Fig. 5*C*). A significantly larger amount of V5-WT than V5-R534∗ was pulled down with Flag-R534∗ AsnRS, confirming the increased heterodimeric interaction between WT and R534∗ compared to homomeric R534∗/R534∗ (Fig. 5*C*).

To further confirm that the R534∗ mutant forms a stable heterodimer with the WT AsnRS, we evaluated heterodimer formation of R534∗ with the endogenous WT AsnRS in the cell. We transiently expressed V5-R534∗ and V5-WT at levels similar to those of endogenous AsnRS. After immunoprecipitation with anti-V5 antibodies, about 3-fold more of the endogenous AsnRS was pulled down by the V5-R534∗ mutant than the V5-WT control (Fig. 5, *D* and *E*), confirming that the WT/R534∗ heterodimer interaction is stronger than the WT/WT interaction in the cell. These results indicate that the WT/R534∗ heterodimer is the predominant dimer form of AsnRS in the cell.

### Substrate binding and catalytic activity may induce destabilization of the dimers

A possible explanation for the discrepancy between *in vitro* and cell-based results for heterodimer formation is the presence of substrates of AsnRS in the cell but not in the *in vitro* analysis. The binding of the substrates triggers enzymatic activity and induces dynamics at the dimer interface to support catalysis. The dominant negative effect of the R534∗ subunit on the WT subunit may reduce these dynamics, thereby effectively stabilizing the heterodimer.

**Figure 6:**
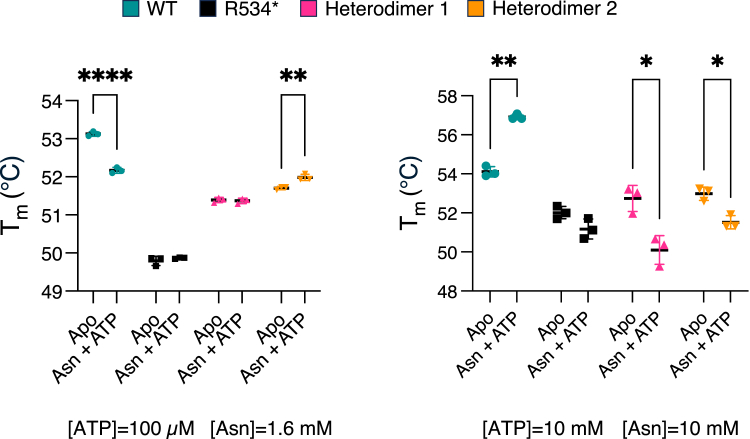
**Melting temperatures of AsnRS WT and R534∗ homodimers and heterodimers determined by thermal shift assay with and without substrates at the indicated concentrations.** N = 3 technical replicates. Two-Way ANOVA with Tukey correction (∗*p*-value < 0.05, ∗∗*p*-value<0.01, ∗∗∗∗*p*-value<0.0001).

Therefore, we monitored T_m_ to probe substrate-induced conformational dynamics. Interestingly, low concentrations of asparagine (Asn; 1.6 mM) and ATP (100 μM) decreased the T_m_ of WT AsnRS, whereas high concentrations of Asn (10 mM) and ATP (10 mM) increased it, presumably due to adenylate formation (Fig. 6). (AsnRS from *E. coli* shows K_m_ values of 15–29 μM for Asn and 76–500 μM for ATP (29), with similar values observed in CHO cells) (30). In contrast, low concentrations of Asn and ATP did not affect the T_m_ of the R534∗ homodimer or the R534∗/WT heterodimers, while high concentrations led to a decrease in T_m_. These results further support the loss of function in the R534∗ homodimer and R534∗/WT heterodimers, and suggest that their impaired activity is associated with reduced substrate-induced dynamics—potentially explaining the discrepancy between *in vitro* and cell-based observations of heterodimer stability.

### R534∗ weakens AsnRS-tRNA binding in the cell

*In vitro* tRNA binding analysis using *in vitro* transcribed tRNA^asn^-GTT transcript and purified AsnRS proteins revealed that the R534∗ mutant exhibited reduced tRNA binding compared to the WT protein in the absence of ATP and Asn (Fig. 3*E*). Addition of ATP and Asn decreased tRNA binding by the WT protein—likely due to tRNA release following aminoacylation—but did not affect the R534∗ mutant (Fig. 3*E*). We also assessed tRNA binding in cells. Concomitant with AsnRS^R534∗^ forming more stable heterodimers with the endogenous AsnRS than AsnRS^WT^ in the cell (Fig. 5*E*), the R534∗ mutant displayed a markedly reduced ability to bind cellular cognate tRNA compared to WT (Fig. 5, *D* and *F*). Together, these results demonstrate that the R534∗ mutation compromises AsnRS–tRNA interactions both *in vitro* and in cells.

## Discussion

*In this study, we have focused on the first reported monoallelic mutation in an aaRS linked to severe neurodevelopmental diseases and symptoms beyond the peripheral nervous system. We have shown that the AsnRS R534∗ mutant is stably expressed in patient cells and* its expression leads to strongly decreased cell proliferation*. Moreover, we have shown that the R534∗ mutant* preferentially forms heterodimers with the WT enzyme, making the *R534∗/WT* heterodimer the predominant form of AsnRS in the cell*. Finally, we demonstrated that the R534∗ mutation not only abolishes enzymatic function within the mutant protomer but also impairs* the activity of the WT subunit when forming a heterodimer. Together, these results establish a dominant-negative loss-of-function mechanism for the AsnRS *R534∗* mutation.

An unexpected feature of the R534∗ mutation is its differential impact on dimerization of AsnRS *in vitro versus* in the cell. While the mutation destabilizes the dimer interface, it still effectively promotes the formation of dimers in the cell. We speculate that dimer stability reflects a balance between interface interactions and conformational dynamics. Interactions at the dimer interface support active site formation, while substrate binding and catalysis require flexibility within the dimer. A mutation can affect dimerization by disrupting the interface or altering active site function. The R534∗ mutation appears to do both, with opposing consequences for dimer stability. It disrupts the dimer interface, reducing the stability of both R534∗/R534∗ homodimers and R534∗/WT heterodimers—more so for homodimers as both subunits are impacted by the mutation (Fig. 5*A*). It also impairs catalytic activity (Figs. 3, *D* and *E*; 4, *B* and *C*), thereby reducing dynamics and paradoxically increasing dimer stability for both dimer types containing at least one mutant protein. When these opposing effects are combined, the resulting dimer stability in the cellular environment ranks as R534∗/WT > R534∗/R534∗ ≈ WT/WT (Fig. 5, *B* and *C*), making the heterodimer the predominant form in cells.

Interestingly, a *de novo* dominant mutation in *SARS1* was also reported to cause a severe neurodevelopmental disorder (31). This mutation results in the insertion of five amino acids (SRWVR) after residue I324 (I324insSRWVR), located near the active site of the enzyme. Patient-derived fibroblasts exhibited reduced SerRS enzymatic activity, and a dominant-negative effect of the mutant SerRS on the WT enzyme was demonstrated using yeast cells. However, the impact of the I324insSRWVR mutation on SerRS homo- and heterodimerization remains uncharacterized. Notably, similar to our observations in *NARS1* R534∗ patient lymphoblasts, fibroblasts from the *SARS1* I324insSRWVR patient also showed severely impaired proliferation and expressed markers of cellular senescence.

Two inherited dominant mutations in *SARS1*, Q239L and S396F, have been reported to cause CMT disease (29). These mutant forms of SerRS exhibit stronger interactions with the WT protein than do WT homodimers (29), making a dominant-negative effect also plausible. Indeed, although the enzymatic defect is milder than that caused by the *NARS1* R534∗ mutation, SerRS activity is nonetheless reduced in patient cells carrying the monoallelic S396F mutation compared to healthy controls. Interestingly, both Q239L and S396F mutations in SerRS are also located near the active site, with neither positioned at the dimer interface. This supports the second scenario proposed above, in which a mutation affects dimerization by altering active site function. The stronger mutant/WT heterodimer interaction is likely not due to a direct effect on the dimer interface but rather a consequence of reduced enzymatic activity and dynamics. If the mutant homodimer is less active than the mutant/WT heterodimer, it may become the predominant dimer form in the cells. This could sequester the mutant protein in the homodimeric state, thereby limiting its ability to poison the WT enzyme. Such a mechanism could explain the relatively mild impact of these mutations on aminoacylation. It would be informative to compare the stability of WT and mutant homodimers *versus* mutant/WT heterodimers for the SerRS variants.

**Figure 7:**
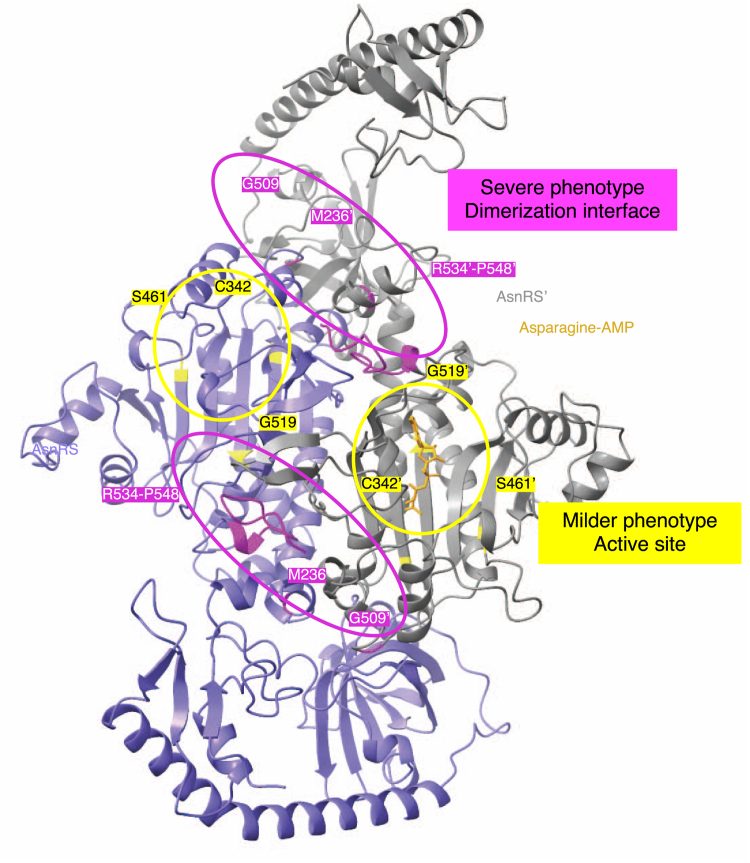
**Localization of neurological disease-causing monoallelic mutations in NARS1.** AlphaFold3 model of *Homo sapiens* AsnRS. R534-P548, M236 and G509 residues (*pink*) linked to severe disease phenotypes are located near the dimerization interface. S461, C342 and G519 residues (*yellow*) linked to milder disease phenotype are located near the active site.

*Monoallelic mutations in AsnRS (e.g., C342Y, S461F, and G519R) can cause CMT and are associated primarily with peripheral neuropathy symptoms* (32, 33)*. In addition to the R534∗ mutation studied here, two other de novo mutations in AsnRS—M236del* (32) *and G509S* (10)*—have been linked to more severe neurodevelopmental phenotypes involving the central nervous system. Unlike the recurrent R534∗* mutation, each of these has so far been reported in only a single patient (10, 11, 32). Interestingly, M236 lies within a short helix near the dimer interface, and deletion of this residue would directly disrupt dimerization (Fig. 7). Even more striking, G509 is positioned just across the dimer interface from this helix (Fig. 7). In contrast, residues associated with pure peripheral neuropathies are located near the active site and distant from the dimer interface (Fig. 7).

Thus, we propose that monoallelic AsnRS mutations can be broadly grouped by their structural proximity to the dimer interface, correlating with distinct clinical presentations. Milder CMT-linked mutations tend to affect the active site without disturbing dimerization directly, while more severe neurodevelopmental cases involve mutations that impact the dimer interface. Both groups impair enzymatic activity *per se*, but the latter group is more likely to exert a dominant-negative effect over the WT enzyme, at least in AsnRS, by making the Mutant/WT heterodimers the predominant form in cells.

*In addition to interfering with WT enzyme function through heterodimer formation, the mutant aaRS may also exert a toxic gain-of-function effect. For example, tRNA sequestration resulting from mutation-enhanced aaRS interaction with tRNA has been proposed as a toxic gain-of-function mechanism in aaRS-linked CMT, and overexpression of the cognate tRNA fully rescued disease phenotypes in animal models of CMT caused by mutations in GlyRS or GARS1* (34). In addition to tRNA, CMT-causing mutant aaRSs have been shown to gain aberrant interactions with molecules unrelated to protein synthesis, which may also contribute to toxicity (4), (35, 36, 37, 38, 39, 40, 41). Interestingly, *we observed reduced AsnRS-tRNA binding by the R534∗ mutation in vitro* (Fig. 3*E*) *and in the cell* (Fig. 5, *D* and *F*)*. This observation contrasts with the enhanced GlyRS-tRNA interaction found in the Gars1*^*C201R/+*^ *CMT mouse model, suggesting a differential disease mechanism. Depending on how catalytically defective the mutant protein is and how the mutant protein is distributed between forming heterodimers with WT or existing independently, the mutation may act predominantly through either a dominant-negative loss-of-function effect or a toxic gain-of-function mechanism. In the case of the R534∗* mutation, the *predominant mechanism is likely to be dominant-negative loss-of-function, and overexpressing the WT enzyme may have a better chance of alleviating the mutant toxicity than overexpressing the cognate tRNA.*

It is important to note that while HEK293T cells provide a useful system for biochemical dissection, they do not recapitulate the tissue-specific context of neurodevelopmental disease. Future studies employing more physiologically relevant models will be essential to elucidate the tissue-specific consequences of this mutation.

## Experimental procedures

### Structure modeling

Structure was represented using the UCSF chimeraX software (42). The structure model was generated using AlphaFold3 (43) by inputting the sequences of human AsnRS and tRNA^Asn^-GTT-1. The atomic coordinates of the asparagine-AMP intermediate were extracted from the crystal structure of human AsnRS (PDB: 8H53) and aligned into the active site of the predicted structure using the matchmaker tool in ChimeraX. Hydrogen bonds were calculated using the H-bond tool and represented in dotted lines.

### Sequence alignment analysis

Protein sequences were retrieved from Uniprot (44) and aligned with Clustal Omega (45). Residue conservation levels are indicated by levels of conservation. R534 and R545 are labeled accordingly.

### Cell culture

#### Patient cells

##### Growth conditions

Lymphoblasts of the patient (P2) and of the father of the patient (P2F) used in previous studies (https://n.neurology.org/content/96/15_Supplement/4714)were EBV immortalized and were cultured in suspension at 37 °C in RPMI medium supplemented with 10% FBS and 1% penicillin and streptomycin. Cells were regularly tested for *mycoplasma* contamination.

##### Western blotting

Cells were lysed using 1x Pierce IP lysis buffer supplemented with 1x protease inhibitor. After 15 min incubation on ice, the lysate was centrifuged for 15 min at maximum speed. The supernatant was boiled for 5 min in 1x SDS with 3% DTT. 5 μl were loaded onto a 4 to 12% sodium dodecyl sulfate (SDS)-polyacrylamide gel (NuPAGE 4–12% Bis-Tris Protein Gels, ThermoFisher Scientific) then ran for 2.5 h at 140 V in MES buffer. The gel was transferred to a nitrocellulose membrane using the iBlot three system. The nitrocellulose membrane was blocked for 1 hour in 5% milk in TBST at room temperature and incubated overnight with a monoclonal antibody recognizing AsnRS1 (anti-rabbit 1:10,000; Abcam, 129,162) at 4 °C. The membrane was washed three times for 5 min in 1% milk in TBST and incubated for 1 h at room temperature with HRP-conjugated rabbit secondary antibody. The membrane was washed three times for 5 min in TBST then visualized using the BioRad imager after incubation in HRP substrate. Anti-alpha-tubulin antibody was used as a loading control (anti-mouse 1:5,000, CST, 3873S).

##### Proliferation assay

Cells were seeded at low density at 10,000 cells/well in a 96 well plate. CCK8 cell viability assay was performed by incubating the cells with 10 μl of CCK8 reagent for 2 h before reading the absorbance at 450 nM. Measurements were repeated daily for 5 days. Data were plotted on GraphPad Prism.

#### HEK293T Cell lines

##### Establishment and selection

HEK293T cells were grown in DMEM medium supplemented with 10% FBS and 1% penicillin and streptomycin. Cells were seeded at day 1 at 167,000 cells per well in six-well plates. The next day (day 2), cells were transfected with 2 μg plasmid using lipofectamine 3000 reagent. The construct intentionally placed the V5-tagged NARS1 gene followed by the EGFP/puromycin fusion gene linked by an IRES (Internal Ribosome Entry Site) element under a shared CMV promoter. This allows the GFP signal to be directly proportional to the V5-AsnRS expression in the cells. On day 4, Cells were treated with 2 μg/ml freshly added puromycin in medium to select for the cells that integrated the transgene successfully. Cells were split cultured until recovery after control cells (non-transfected) died. The NARS1-WT plasmid was purchased through VectorBuilder and mutations were cloned using the ligation independent cloning method.

##### FACS: two different populations selected

Since AsnRS WT or mutant influenced cell viability, cells expressing low or high expression of GFP were sorted at day 15 to comparable expression of plasmid in each cell line and subsequently cultured in the appropriate vessel. After sorting, 21,000 cells for each construct with high GFP expression were seeded in a 48-well plate and 100,000 cells each construct with low GFP expression were seeded in a six-well plate. The flow cytometry results were analyzed using FlowJo at day 18 and day 26.

##### Pictures and cell quantification

Pictures of live low- and high-GFP expressing cells were taken directly into 48 well plate were taken at 1, 2 and 3 days after seeding. Pictures were taken using a microscope on bright field and GFP, 4x and 20x magnification. For 20x magnification, the same field of view was used for bright field and GFP. Cells were then quantified manually using the 20x bright field images and plotted using the GraphPad Prism software.

##### Western blotting

Cells were recovered after 4 and 14 days in culture post-puromycin selection and lysed in 1x buffer with protease inhibitor. 15 μg total protein was loaded onto an acrylamide gel and ran for 1 h. Western blot was prepared and visualized as described previously.

#### Transient transfection in HEK293T and co-immunoprecipitation

167,000 HEK293T cells were seeded per well in a six-well plate. The next day, cells were co-transfected with 1 μg plasmid per construct per well using lipofectamine 3000 reagent, where co-transfected plasmids were incubated together before being added to the cells. 48 hours after transfection, cells were lysed in 500 μl 1x RIPA buffer supplemented with protease inhibitor. After 15 min incubation on ice, cells were centrifuged for 15 min at max speed. In the meantime, 5 μl of magnetic beads per sample were incubated for 1 hour with 500 ng Flag antibody (CST, 2368S) or IgG rabbit control. 50 μl of cell lysate supernatant was collected for western blotting to measure Flag, V5 and AsnRS input in total protein. Flag and IgG coated beads were then washed and resuspended in 350 μl cell lysate supernatant and incubated overnight for IP alone part. The next day, beads were washed with 500 μl 1x RIPA buffer with protease inhibitor three times for 7 min each. Supernatant was removed, and beads were resuspended directly in 50 μl 1x SDS and boiled for 5 min, vortexed, boiled for another minute. 10 μl was loaded for Flag control and 20 μl was loaded for V5 signal. Gel ran for 2 hours at 140V in MES buffer and was processed like previously described using BSA or milk for blocking, then incubated with primary antibodies against Flag (CST, 8146S, 1:5000), V5 (Invitrogen, 46–1157, 1:5000) and AsnRS. Fluorescent secondary antibodies (LI-COR) were used to detect Flag, V5 and AsnRS bands. Data were plotted in GraphPad Prism and samples were compared using a one-way ANOVA statistical test. R545S mutant was cloned and added to control for the signal in IgG binding of R545C mutant.

### Coimmunoprecipitation-northern blot

Protocol was adapted from a previously published protocol (46). Each condition was repeated in technical triplicates. Previously described transfected HEK293T cells were sorted by FACS for GFP expression so that plasmid expression in cell lysate would match endogenous AsnRS expression and be equivalent from one construct to another. Confluent 15-cm plates of cells were washed with ice-cold PBS, then covered with 5 ml ice cold PBS before UV crosslinking. Cells were crosslinked twice for 2 min using a UV crosslinker. Cells were scraped off the plate, spun down for 5 min at 4000*g* then resuspended in 800 μl Pierce IP lysis buffer supplemented with 1x protease inhibitor (1x) and RNAse inhibitor. From there, all the steps were performed in RNAse free conditions and at 4 °C. After 15 min incubation on ice, cells were centrifuged for 15 min at max speed. In the meantime, 5 μl of magnetic beads per sample were incubated for 1 h with 40 μg V5 antibody or IgG control in 1x Pierce IP lysis buffer with 1x protease inhibitor and RNAse inhibitor. 50 μl of cell lysate supernatant was collected for western blotting to measure V5 and AsnRS input in total protein. V5 and IgG coated beads were then washed and resuspended in 350 μl cell lysate supernatant and incubated overnight for IP alone part. For IP-northern blot, 700 μl of the rest of the supernatant was incubated with V5-coated beads. The next day, beads were washed with 500 μl wash buffer (46) with protease and RNAse inhibitor two times for 5 min each. Beads were resuspended in 500 μl wash buffer. 50 μl was set aside for western blotting to measure IP input for V5 and AsnRS. For protein input, supernatant was removed, and beads were resuspended directly in 50 μl 1x SDS and boiled for 5 min, vortexed, boiled for another minute. 20 μl supernatant was used for Western blotting. Gel ran for 2 h at 140V and processed like previously described using 5% BSA. Fluorescent antibodies were used to detect V5 and AsnRS bands. 250 μl Trizol was added to the remaining 450 μl beads after removal of the supernatant and RNA were extracted using the Trizol extraction protocol. Final RNA pellet was resuspended in 5 μl RNAse-free water and 5 μl 2x RNA loading dye. 10% urea gel was loaded with 10 μl RNA mix or 1ng *in vitro* transcribed tRNA after a 30-min empty pre-run. Gel ran for 45 min at 240 V in 1x TBE. The gel was then incubated for 15 min in 1x SYBR gold in 1x TBE, imaged, then transferred onto a nylon membrane for 1 h at 5V. The nylon membrane was UV crosslinked, blocked for 30 min in hybridization buffer and incubated with a custom 5′ labeled biotin tRNA^Asn^-GTT probe (IDT, sequence: CG TCC CTG GGT GGG CTC GAA CCA CCA ACC TTT CGG TTA ACA GCC GAA CGC GCT AAC CGA TTG CGC CAC AGA GAC) or 3′ labeled biotin tRNA^Ser^-GCT (GAC GAG GRT GGG ATT CGA ACC CAC GYG TGC AGA GCA CAA TGG ATT AGC AGT CCA TCG CCT TAA CCA CTC GGC CAC CTC) overnight at 60 °C in hybridization buffer (20 mM Sodium Phosphate pH 7.2, 300 mM NaCl, 1% SDS). Membrane was washed twice in northern blot wash buffer (20 mM Sodium Phosphate pH 7.2, 300 mM NaCl, 0.1% SDS, 2 mM EDTA) for 10 min each at room temperature, incubated with HRP-conjugated streptavidin antibody (BioLegend, CAT: 405210, 1:2000) for 30 min at 37 °C in hybridization buffer. Membrane was washed 3 times in wash buffer for 5 min at room temperature and incubate with chemiluminescent substrate before visualization on BioRad imager. Signal was quantified using the BioRad quantification software. Values were normalized over total protein input for protein and total RNA from IP SYBR gold staining for RNA. Conditions were compared using graphPad Prism and an unpaired Welch’s statistical *t* test.

### Recombinant AsnRS homodimer purification

#### Cloning

Total RNA was extracted from HEK293T cells using Trizol. The human NARS1 WT coding sequence was reverse transcribed using the Superscript-RT kit, purified and cloned into a pET-28a vector (Novagen) carrying a N-terminal 6xHis tag and the Kanamycin resistance gene. R534∗ (1600 C > T) and R545C (1633 C > T) mutations were cloned into the vector using the ligation independent cloning method. Sequences were confirmed by sanger sequencing.

#### Cell expansion and purification

Constructs were transformed into BL21(DE3) or BL21 ClearColi cells by heat shock or electrophoresis. Cultures were grown overnight to saturation in LB medium containing 50 μg/ml Kanamycin or 25 μg/ml Ampicillin. The overnight culture was diluted 1/100 in LB medium and grown at 37 °C. Isopropyl ß-D-thiogalactopyranoside (IPTG) was added to a final concentration of 1 mM at OD600 of 0.7, and then the cells were grown at 16 °C overnight. The cells were collected by centrifugation at 4,000 rpm for 30 min.

For 6xHis tagged constructs, the pellet was resuspended in lysis buffer (20 mM Tris-Cl pH 8.0, 500 mM NaCl, 5 mM Imidazole, 5 mM β-Mercaptoethanol (BME), 10% glycerol). The cells were disrupted by an M110L Microfluidizer (Microfluidics) and the lysate was clarified by centrifugation at 28,000 rpm for 30 min. AsnRS and all the recombinant mutant proteins were purified using Ni-NTA beads (Thermo Fisher Scientific). To remove residual Asn-AMP from AsnRS, the proteins were treated with 0.5 M Hydroxylamine for 30 min on ice before dialysis into a low-salt buffer (Buffer A: 20 mM Tris-HCl, pH 7.4, 100 mM NaCl, 5 mM BME, 5% Glycerol). The proteins were then loaded on a HiTrap Heparin HP column (Cytiva) and eluted with a salt gradient from Buffer A to a high-salt buffer (Buffer B: 20 mM Tris-HCl, pH 7.4, 1 M NaCl, 5 mM BME, 5% Glycerol) followed by size exclusion chromatography with a HiLoad 16/60 Superdex 200 prep grade column (Cytiva). All purification steps were carried out at 4 °C or on the ice. Purified protein was stored at −80 °C in 150 mM Tris-HCl pH 7.4, 100 mM NaCl and 5 mM BME.

For STREP-II tagged constructs, imidazole was removed from the initial buffer and the Ni-NTA beads were replaced by a Strep-Tactin Sepharose column (1 ml) and eluted with desthiobiotin according to the manufacturer’s instructions.

### Recombinant AsnRS heterodimer purification

Recombinant heterodimers and controls were purified according to our published protocol (28).

### Thermal shift assay

Thermal shifts assays were performed on a StepOnePlus 96 Real Time Cycler (Applied Biosystems). The dye from the Protein Thermal Shift Kit (Thermo Fisher Scientific) was used to monitor the thermal stability of protein by binding to the exposed hydrophobic regions. Solutions of 5 μl of protein thermal shift buffer, 2.5 μl of diluted thermal shift dye (8X) and 12.5 μl of protein at 0.1 mg/ml final concentration were added to the wells of a 96-well Optical Reaction Plates (Applied Biosystems). Freshly prepared asparagine and ATP (at the concentrations indicated in Fig. 6) were added to the reaction mix. The plates were sealed with optical sealing tape (Bio-Rad) and heated with the RT-PCR from 25 to 90 °C in increments of 0.5 °C. Measurements were done in triplicate and analyzed with the corresponding Protein Thermal Shift Software. The fluorescence signal is monitored and plotted *versus* the temperature and the midpoint of the protein unfolding transition is defined as the Tm. Results were compared using the corresponding one-way or two-way ANOVA statistical test.

### *In vitro* tRNA transcription

tRNA^Asn^-GTT was obtained by *in vitro* transcription using T7 RNA polymerase according to standard protocols (https://pubmed.ncbi.nlm.nih.gov/27386510/). The DNA sequence for tRNA^Asn^-GTT-2 was cloned into a pUC-19 vector under the control of a T7 RNA polymerase promoter. DNA-templates for *in vitro* transcription were amplified by PCR using forward and reverse primers complimentary to the T7 promoter and the 3′ end of the tRNA gene, respectively. Transcription reactions were performed in 40 mM Tris–HCl pH 8.0, 25 mM NaCl, 25 mM MgCl_2_, 2 μg/ml yeast pyrophosphatase (Roche), 1 mM Spermidine, 5 mM DTT, 18 mM GMP, 4 mM each of ATP, CTP, GTP and UTP with 75 μg/ml T7 RNA polymerase and DNA template at 37 °C for 6 h. Reactions were stopped by phenol/chloroform extraction followed by purification of the tRNA by 12% denaturing PAGE. tRNA was eluted from the gel in buffer containing 200 mM NaOAc, 20 mM Tris–HCl, 5 mM EDTA (pH 5.3), followed by ethanol-precipitation. The final tRNA was taken up in RNAse-free water and stored at −80°C. The purified tRNA was annealed by heating up to 95 °C for 3 min and then slowly cooled down to room temperature by adding 1 mM MgCl_2_ at 55 °C.

### Ni-NTA pull down assay to assess AsnRS-tRNA interaction

Binding reactions (200 μl) were prepared containing binding buffer (25 mM HEPES pH 7.5, 20 mM KCl, 5 mM MgCl_2_, 5 mM Imidazole, 0.2 mM DTT), 1 μM *in vitro* transcribed tRNA^Asn^-GTT-2, and 2 μM of either wild-type (WT) or R534∗ 6xHis-tagged AsnRS. Reactions were supplemented with or without 10 mM ATP and 10 mM asparagine and incubated for 30 min at room temperature. A control reaction without AsnRS was included to assess non-specific tRNA binding.

Following incubation, input samples were collected. Subsequently, 30 μl of a 50:50 Ni-NTA bead (Thermo Fisher Scientific) slurry, pre-washed and resuspended in binding buffer, was added to each reaction. Samples were incubated at room temperature on a rotator for an additional 30 min. The Ni-NTA beads were pelleted by centrifugation at 700*g*, and supernatants were removed. Ni-NTA bead pellets were washed twice with 250 μl of wash buffer (25 mM HEPES pH 7.5, 20 mM KCl, 5 mM MgCl_2_, 20 mM Imidazole, 0.2 mM DTT). For reactions initially treated with substrates, the wash buffer also contained 10 mM ATP and 10 mM asparagine.

After decanting the wash buffer, Ni-NTA bead pellets were resuspended in 100 μl of PBS and transferred to tubes containing either 2x SDS or 2x RNA loading dye (New England Biolabs). Samples were boiled for 5 min, centrifuged, and loaded onto 4 to 12% Bis-Tris or 10% TBE-Urea polyacrylamide gels (Thermo Fisher Scientific). Gels were stained with silver stain (Bio-Rad) or SYBR Gold (Thermo Fisher Scientific) and imaged using a Bio-Rad ChemiDoc MP. Densitometric analysis was performed using Bio-Rad Image Lab software.

### Mass photometry

AsnRS Dimerization *K*_d_ was measured using mass photometry. Enzymes concentrations were determined using a BCA kit and were diluted to working concentrations before the measurements, between 5 and 100 nM. Counts in monomer and dimer peaks were added to get the total counts. Dimer concentration was calculated according to previously described protocols (47, 48). *K*_d_ averages were compared using the one-way ANOVA statistical test with Tukey correction.

### Active site titration ATP hydrolysis assay

The active site titration assays were performed at room temperature with 100 mM HEPES pH 7.5, 20 mM KCl, 5 mM MgCl2, 25 μM cold ATP, 1 mM DTT, 4 μg/ml pyrophosphatase, 10 mM freshly prepared L-Asparagine, 0.03 μM γ-radiolabeled ^32^P-ATP (3000 Ci/mmol, 10 mCi/ml, BLU502A250UC, Revvity Health Sciences) as the experiment buffer. The protocol was adapted from a previous study (49). The reaction was initiated by adding various concentrations of AsnRS protein into the mixture and various concentrations of *in vitro* transcribed tRNA^Asn^ GTT-2 were added. At varying time intervals, 5 μl aliquots were applied to MultiScreen 96-well filter plate (0.45 um pore size Hydrophobic, low protein binding membrane (Millipore), which is pre-wetted with 80 μl of quench solution containing 10% charcoal slurry in 0.5% HCl in 7% HClO_4_. After the time points were collected, the plate was centrifuged into a 96-well flexible PET microplate (PerkinElmer) with 150 μl of Supermix scintillation cocktail (PerkinElmer). After mixing and adding 2.5 2x solution for total activity, the radioactivity in each well of the plate is counted in the 1450 LSC & Luminescence Counter (PerkinElmer).

### Aminoacylation

Purified proteins were incubated in triplicate at 37 °C for 3 min in a reaction buffer (50mM Tris-buffer (pH 7.5), 12mM MgCl_2_, 25mM KCl, 1 mg/ml bovine serum albumin (BSA in PBS), 0.5mM Spermine, 1mM ATP, 0.2mM yeast total tRNA, 1mM Dithiothreitol, 0.3 mM [^15^N_2_]-asparagine (freshly prepared). The reaction was terminated using trichloroacetic acid (TCA). After termination with TCA, ammonia was added to release the labelled amino acid from the tRNAs. [^13^C_2,_^15^N]-glycine was added as internal standard and the labelled amino acids were quantified by LC-MS/MS (50). Results depicted in Figure 3*F* were obtained using 1.5 μM of AsnRS, during a 10 min assay. while data depicted in Figures 3*G* and 4*C* and S3*B* was obtained using 0.375 μM of AsnRS during 3 min incubation. Results were compared using unpaired T-tests or one-way ANOVA statistical tests, respectively.

## Data availability

The authors confirm that the data supporting the findings of this study are available within the article and its supporting materials.

## Supporting information

This article contains supporting information.

## Conflict of interest

The authors declare that they have no conflicts of interest with the contents of this article.
