# Supplementary material for: Dominant-negative NARS1 R534∗ mutation causes wild-type subunit poisoning and heterodimer predominance in cells
Source: J Biol Chem. 2025 Sep 4;301(10):110690. doi: 10.1016/j.jbc.2025.110690 (PMC12513289; doi:10.1016/j.jbc.2025.110690)
Supplement: Figures S1–S3 [file mmc1.pdf]

Supporting Figure S-1

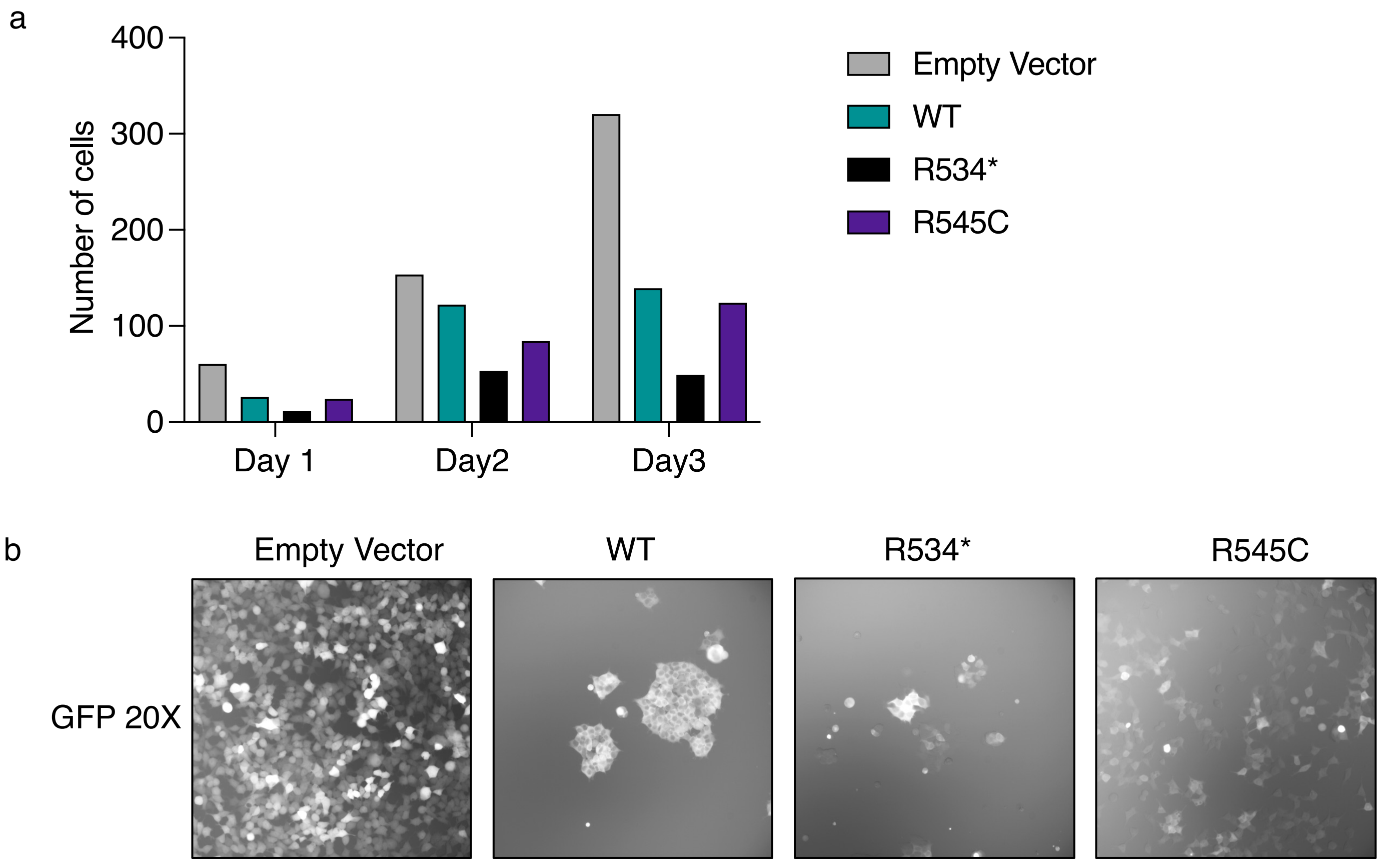

Supporting Figure S-1: The R534* mutant shows dominant toxicity in cells
HEK293T cells are transfected with constructs expressing empty vector (EV), V5-AsnRS WT, R534* or R545C and selected for higher or lower GFP expression. (a) Quantification of cell numbers at day 1, 2 and 3 after high or low GFP selection by FACS, N=1. (b) GFP image of cells in culture at day 3 after selection.

Supporting Figure S-2

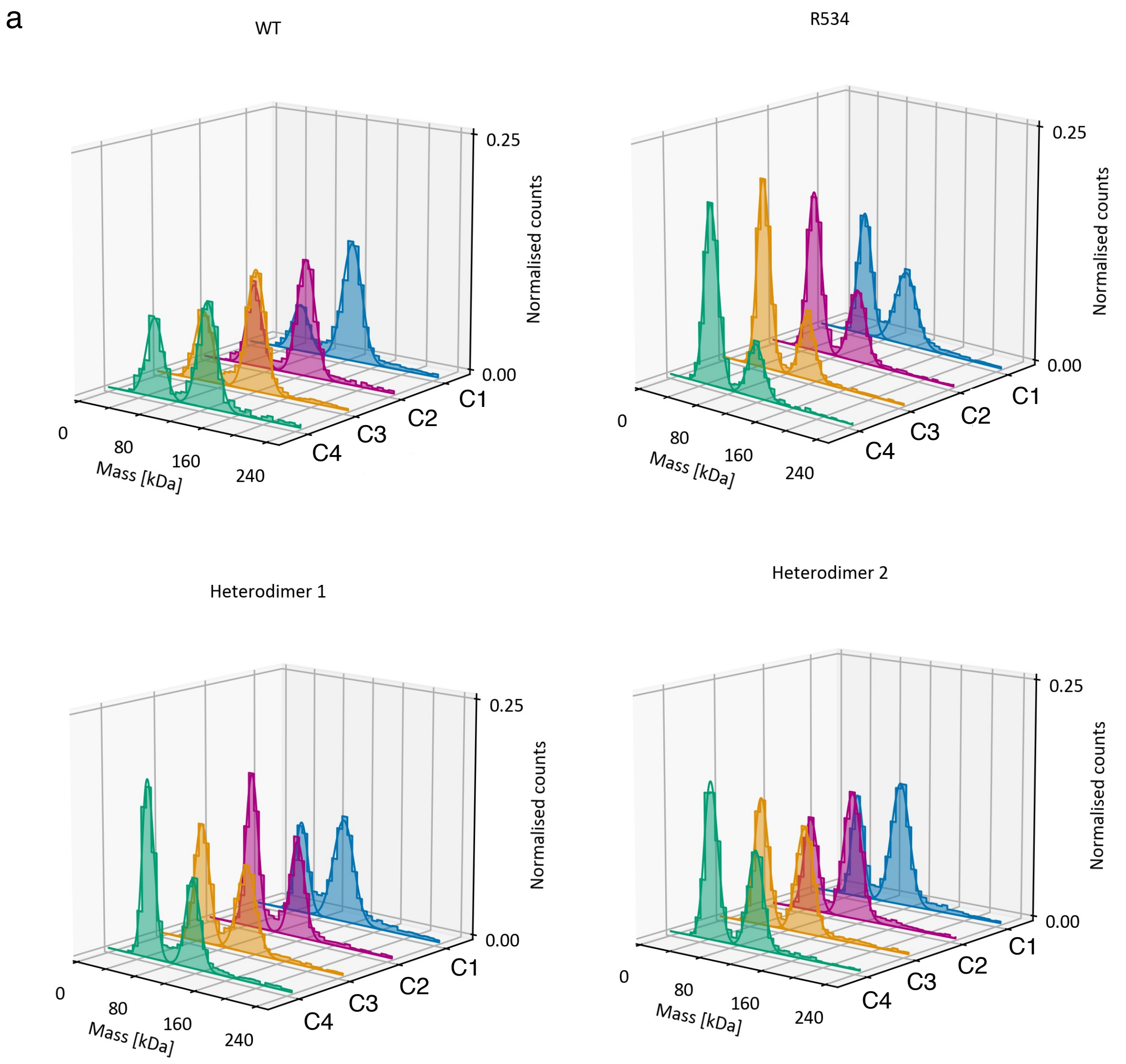

b

	[monomer] (nM, corrected)				[dimer] (nM, corrected)			
Input concentration (nM, corrected)	C1	C2	C3	C4	C1	C2	C3	C4
WT	11.9	6.6	4.8	2.9	17.0	5.5	4.3	2.1
R534*	34.7	18.8	15.1	8.1	15.6	5.7	2.9	1.4
Heterodimer 1	29.3	16.9	12.5	9.2	24.9	6.1	5.7	2.7
Heterodimer 2	26.4	15.6	10.4	6.4	24.0	12.9	5.1	2.2

Supporting Figure S-2: Mass photometry profiles of titration of AsnRS to calculate dimerization K_d

(a) 3D histograms of mass photometry measurements of AsnRS WT, R534*, heterodimer 1 and heterodimer 2 with titrated concentrations. Four concentrations measured from highest to lowest: C1, C2, C3 and C4 nM. (b) Table: Concentration of monomer and dimer in nM calculated from counts in monomer (64 kDa) and dimer (128 kDa) peaks.

Supporting Figure S-3

a

	WT (10 µM)	WT (10 µM) + tRNA (10 µM)	R534* (10 µM)	R534* (10 µM) + tRNA (10 µM)	R545C (10 µM)	R545C (10 µM) + tRNA (10 µM)
Slope	0.5	0.6	0.2	0.3	0.9	1.1
Y-intercept	3.1	4.7	0.1	-0.004	1.0	1.6

b

	WT (8 *µ*M)	WT (8 *µ*M) + tRNA (8 *µ*M)	R534* (8 *µ*M)	R534* (8 *µ*M) + tRNA (8 *µ*M)	No enzyme control
Slope	0.6	0.7	0.05	0.05	-0.008
Y-intercept	2.1	3.4	-0.2	-0.2	0.1

c

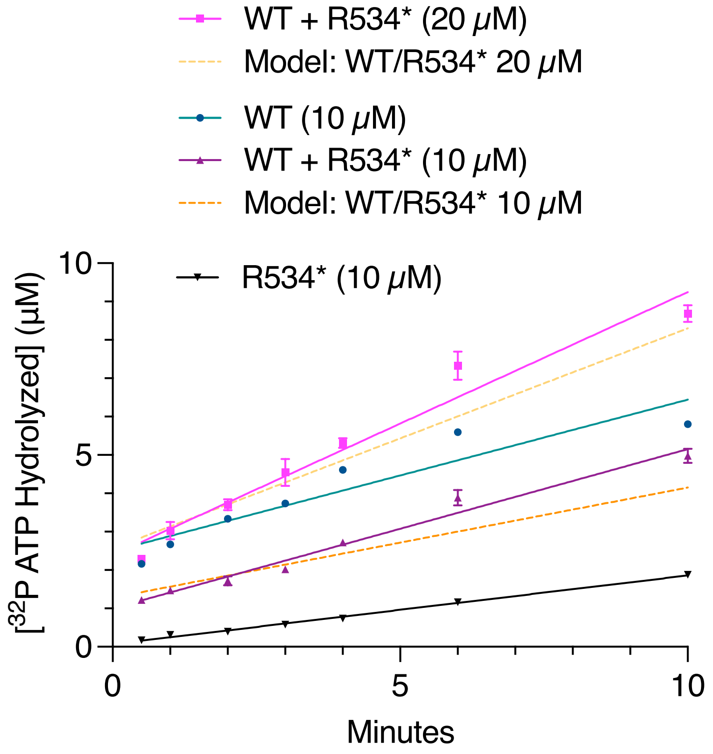

d

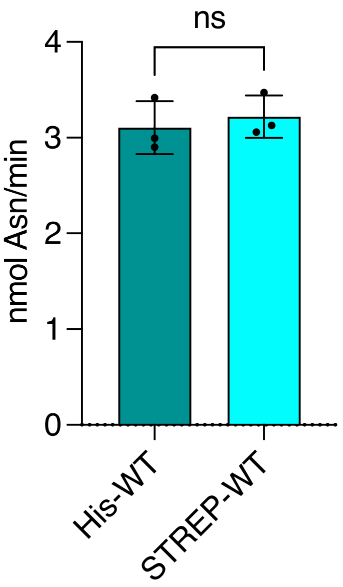

Supporting Figure S-3: AsnRS enzymatic activity analyses

(a-b) Simple linear regression analysis of the ATP hydrolysis assay shown in Fig. 3c-d, respectively. The slope and Y-intercept were calculated from the fitted data. (c) ATP hydrolysis activity of WT, R534* homodimers and equimolar mix of WT and R534* homodimers (10 µM or 20 µM). The model activity of WT/R534* was calculated as (WT+R534*)/2. (d) Aminoacylation activity of recombinant His-WT and STREP-WT at 375 nM after a 3-minute incubation. Unpaired T-test indicates no significant difference (ns). N=3 technical replicates.
